# Clinicopathologica Epidemiological Characteristics and Change Tendencies of Renal Cell Carcinoma in Shanxi Province of China from 2005 to 2014

**DOI:** 10.1371/journal.pone.0144246

**Published:** 2015-12-03

**Authors:** Tao Bai, Li Wang, Dongwen Wang, Xiaobin Yuan, Wei Bai, Qin Yang, Xian Yang

**Affiliations:** 1 Department of Urology, The First Hospital of Shanxi Medical University, Taiyuan, Shanxi, People's Republic of China; 2 School of Public Health, Shanxi Medical University, Taiyuan, Shanxi, People's Republic of China; 3 Department of Pathology, Shanxi Cancer Hospital, Taiyuan, Shanxi, People's Republic of China; 4 Department of Pathology, Shanxi Province People's Hospital, Taiyuan, Shanxi, People's Republic of China; 5 Department of Pathology, The First Hospital of Shanxi Medical University, Taiyuan, Shanxi, People's Republic of China; University of Texas Health Science Center San Antonio Texas, UNITED STATES

## Abstract

**Objectives:**

RCC is the most common solid renal malignancy in adults worldwide. To provide the insight of clinicopathologica epidemiological characteristics and change tendencies of renal cell carcinoma (RCC), 2154 cases were collected from Shanxi Province of China, including diagnose time, age, gender, tumor size, Fuhrman grade, tumor stage, tumor location, local advance or distant metastasis and first symptom from 2005 to 2014. This retrospectively investigation, as its general objective, was to analyze the clinicopathologica epidemiological characteristics and the change tendencies of RCC.

**Methods:**

Between 2005 and 2014, 2154 patients who were diagnosed with RCC in three large tertiary hospitals at Shanxi Province were included. The patients’ demographic features, pathological diagnoses and metastatic statuses were analyzed. Statistics methods include the chi-squared test, analysis of variance, Spearman’s correlation analysis, Logistic regression and ARIMA modeling.

**Results:**

Of the 2154 included patients, the constituent ratio of female /male was 63.1% and 36.9%, with the median age of 57 years old. Fuhrman grade distributions differed significantly between males and females (*p* = 0.024). Also, a significant difference in tumor size was found by different clinical stages (*p* < 0.001), with a linear correlation (*p* < 0.001). Moreover, Spearman’s analysis indicated tumor grade has a negative correlation with female (*p* = 0.009) and a positive correlation with tumor size (*p* = 0.000). It was found that the tumor diameter is bigger in the left side (*p* = 0.022). Furthermore, the metastasis rate was higher in the bigger tumor (*p* < 0.001) and the left-sided tumors (*p* = 0.027). Logistic regression also showed that tumor size is a risk factor for metastasis (OR = 1.724). The risk of local advance or distant metastasis in the left kidney was 1.6-fold greater than that of the right kidney. From 2005 to 2014 the number of RCC cases gradually increased (mainly for pathological grade II and III, but grade I and IV), while the average tumor size decreased, showing the severity increase mildly. Base on the results of a time series analysis-prediction the average RCC size would continue to decrease from the first quarter of 2015 to the fourth quarter of 2016.

**Conclusions:**

The cases of RCC increased from 2005 to 2014 with clear cell type as the main pathological type in this population. The characteristics in the constituent ratios of the RCC vary depending on gender, pathological grade, tumor size, and location, which may be the important factors impacting treatment and prognosis.

## Introduction

The incidence of renal carcinoma (RC) ranks first among malignant tumors of the urinary system, while the incidence of renal cell carcinoma (RCC) ranks second among malignant tumors of the urinary system only following bladder carcinoma in China. According to recent literature from the International Agency for Research in Cancer, the incidence of renal cell carcinoma (RCC) has increased worldwide over the last few decades, particularly in males. In the United States, the incidence and mortality of RCC have been climbing annually since 1950, increasing by 126% and 36.5%, respectively, while the survival rate has been improved only by approximately 9% [1]. Especially, the incidence of RCC has increased more rapidly in recent years, at a rate of 2.85% annually [2]. The incidence of RCC is also growing in the majority of Asian countries. For instance, in China, RCC is more common in males, with an average increase rate at 7.6% per year. Hong Kong and Shanghai are the most affected areas in China. However, the incidence of RC in females is the highest in India, increasing by 2.0% per year, in particular Chiang Mai and Mumbai are the worst areas [3]. Statistics data from the Chinese National Cancer spectrum show that the incidence of RC has increased by 6.5% per year over the past 20 years and with 40% of patients dying from RCC. Because approximately 20%–30% of RCC patients received a terminal cancer diagnosis upon their first visit to the hospital and approximately one-third of patients presented recurrent or metastasis cancer and this cancer were associated with more than 140,000 deaths per year [4]. Therefore, studying pathological characteristics and changes of RCC will provide new insights for understanding the pathology trend of RCC and help for developing new strategy in improving clinic prognosis and therapy. Our research team first investigated the distribution of RCC case in 8 tertiary hospitals in Shanxi Province of China. We found that most cases of the RCC with completed diagnoses and treatments records were concentrated on the three large general hospitals, which covers 80% of total cases. We therefore chose these 3 hospitals out of the 8 for case data collection. Although there are 120 cities and counties in Shanxi Province, the patients in these three hospitals came from 106 cities and counties in Shanxi (covering 88.3%). This retrospectively investigation, as its general objective, was to analyze the clinicopathologica epidemiological characteristics and change tendencies of RCC.

## Methods

### Cases collection

This study was conducted in three largest hospitals of Shanxi Province. A total 2154 cases were obtained with RCC diagnosed between 2005 and 2014. We examined the pathological results and whether metastasis occurred in these case records. Also we collected detail information of patients (age and gender) and the clinical record (diagnose time, tumor size, Fuhrman grade, tumor stage, tumor location, local advance or distant metastasis and the symptom of the first clinic visit). The information was organized on a data collection sheet designed for this study, where the variables were specified. The data has been collected by two investigators separately. The other investigators assessed the integrity and accuracy of the information by double checking. All investigators were trained by a consistent method at the same time, including the diagnostic criteria of the RCC, the standard methods for recording information. All clinical case record of the RCC collected for this study was approved by the Internal Review Board of these hospitals.

We collected a minimum of five pathology sections per renal mass for a total of ten sections per person, regardless of the presence or absence of morphologically demonstrable lesions. The paraffin-embedded tissue sections were assessed following hematoxylin and eosin staining. After a complete review, the histological types were categorized as follows: clear cell, papillary, chromophobe, mixed, RC associated with Xp11.2 translocations/TFE3 gene fusion, tubulocystic carcinoma, etc. The tumor grade was categorized by the Fuhrman grade (I-IV), and the staging (T1, T2, T3, T4, N1, and M1) was determined based on pathological findings according to the American Joint Committee on Cancer (AJCC). Dividing into incidence of renal cell carcinoma group and non-incidence of renal cell carcinoma group according to whether the patients had symptom or not when their first visit.

### Ethics statement

The study was approved by the Ethics Committee for Medicine of the First Hospital of Shanxi Medical University, PR China. All records of patient and information were anonymized and de-identified prior to analysis.

### Statistical analysis

This information (diagnose time, age, gender, tumor size, Fuhrman grade, tumor stage, tumor location, local advance or distant metastasis and first symptom) was input into an Excel sheet. All statistical analyses were performed by using SPSS v13.0 statistical software. Descriptive analysis of the data was presented as percentage, mean and standard error. Qualitative data (percentage of clinic stage) were compared between Fuhrman grade by using the chi-squared test; qualitative data (detection rates of RCC local advance or distant metastasis) were compared between different groups determined by tumor location and clinic stage by using the chi-squared test; while the quantitative data (tumor size) were compared between different Fuhrman grade groups and different years by using one way analysis of variance (ANOVA). Furthermore, associations between each pathological grade, size of tumor and gender were assessed using Spearman’s correlation analysis. And a binary classification logistic regression analysis was used to assess the risk factors of tumor metastasis. We also used an auto-regressive integrated moving average (ARIMA) modeling of the time series analysis to predict future tumor sizes.

## Results

### General situation

Of the 2154 included patients, 63.1% (1360) were men, 36.9% (794) were women, and with the median age was 57 yrs old. Among all age groups, the 50–60 yr group contained the greatest proportion of patients (34.4%). As for tumor location, all cases were unilateral, and 52.3% (1127) cases occurred on the left side, and 47.7% (1027) cases occurred on the right side. 55.2% (1189) patients were incidental renal carcinoma found by annually physic examination, 44.8% (965) patients were non-incidental renal carcinoma (Table 1).

**Table 1:** Distribution of the 2154 cases.

Group	Number	Percentage (%)
**Age(years)**		
<30	35	1.6
30-	108	5.0
40-	424	19.7
50-	741	34.4
60-	576	26.8
70-	268	12.5
**Tumor location**		
Left side	1127	52.3
Right side	1027	47.7
**Symptom**		
Incidental renal carcinoma	1189	55.2
Non-incidence renal cell carcinoma	965	44.8
**Histological type**		
Clear cell type	1887	87.6
Papillary	63	2.9
Chromophobe	61	2.8
Mixed	29	1.3
Renal carcinoma associated with Xp11.2 Translocations/TFE3 gene fusion	6	0.3
Tubulocystic carcinoma	5	0.2
Unclassified	103	4.8

### The characteristics in the constituent of RCC pathological grade

Histological types of RCC showed a predominance of the clear cell type (1887, 87.6%), followed by lower proportions of the papillary (63, 2.9%) and chromophobe (61, 2.8%) types (Table 1).

Only 1315 out of 2154 cases were with clearly record of Fuhrman grades. These cases of RCCs were further classified as grade I (63, 4.8%), grade II (845, 64.3%), grade III (360, 27.4%) and grade IV (47, 3.6%). The result from *x* ^*2*^ test indicated that the Fuhrman grade distributions differed significantly between males and females (*x* ^*2*^ = 9.461, *p* = 0.024). In addition, 70.36% were grade I and II in incidence renal cell carcinoma group. Statistically significant difference in Fuhrman grading was found among incidence renal cell carcinoma group and non-incidence renal cell carcinoma group (*x* ^*2*^ = 42.636, *p* = 0.000) (Table 2).

**Table 2:** Percentage distribution by the Fuhrman grade.

Group	Number of Fuhrman grade (%)				*x* ^*2*^	*p*
	I	II	III	IV		
**Gender**						
Male	32 (3.8)	529 (63.3)	239 (28.6)	36 (4.3)	9.461	0.024
Female	31 (6.5)	316 (66.0)	121 (25.3)	11 (2.3)		
**Symptom**						
Incidental renal carcinoma	22 (4.6)	294 (61.9)	121 (25.5)	38 (8.0)	42.460	0.000
Non-incidence renal cell carcinoma	41 (4.9)	550 (65.5)	240 (28.6)	9 (1.1)		
**Clinical stage**						
T1a	37 (63.8)	408 (54.9)	123 (37.3)	9 (20.9)	71.808	0.000
T1b	16 (27.6)	248 (33.4)	122 (37.0)	19 (44.2)		
T2a	5 (8.6)	63 (8.5)	53 (16.1)	12 (27.9)		
T2b	0 (0.0)	24 (3.2)	32 (9.7)	3 (7.0)		

Note: The Fuhrman grade and clinical stage were recorded simultaneously for 1174 cases.

In 1375 cases recorded the largest tumor diameter; the tumor size ranged from 0.70 cm to 28 cm, with an average size of 5.10 ± 2.792 cm. As for the clinical stage, they were divided into T1a (664, 48.29%), T1b (479, 34.84%), T2a (115, 11.27%) and T2b (77, 5.60%). A significant difference was found in the distribution of clinical stages according to Fuhrman grades (*x* ^*2*^ = 71.808, *p* = 0.000) (Table 2), indicating a linear correlation (*x* ^*2*^ = 54.053, *p* = 0.000). Moreover, the average tumor size differed significantly by Fuhrman grade (*F* = 25.013, *p* = 0.000) (Table 3).

**Table 3:** Average size of tumor by the Fuhrman grade.

Fuhrman grade	Number	Mean ± S.E.	*95%CI*	*F*	*p*
I	58	4.214 ± 0.227	(3.760, 4.668)	25.013	0.000
II	743	4.623 ± 0.085	(4.456, 4.790)		
III	330	5.829 ± 0.169	(5.496, 6.162)		
IV	43	6.695 ± 0.077	(5.767, 7.623)		

### Correlation analysis between the tumor size, grade and gender

To find out the association between gender and tumor grating, Spearman’s analysis was carried on. The results indicated a negative correlation between Fuhrman grade and female gender (*r* = -0.072, *p* = 0.009), i.e. the RCC pathological classification was lower in female than that in male. A positive correlation was found between tumor size and the Fuhrman grade (*r* = 0.226, *p* = 0.000), and a negative correlation was found between the size of tumor and the right side (*r* = -0.062, *p* = 0.022), namely, the size of tumor was smaller in right side; while the bigger RCC tumor size, the higher grade.

### Renal cell carcinoma local advance or distant metastasis

Among all 2154 cases, 99 exhibited local advance or distant metastasis (4.6%). Local advance includes the local infiltration and regional lymph nodes metastasis. In the 99 cases, 53.5% of the total cases advance to stage T3; 22.2% cases advance to stage T4; 20.2% cases in stage N1 were detected with regional lymph nodes metastasis, including retroperitoneal lymph nodes, para-aortic lymph nodes and renal hilum lymph nodes; and 13.1% cases in stage M1 being distant metastasis to several areas, such as the peritoneum, costophrenic angle, vertebral body, hilus of spleen, and ovaries. By using AJCC kidney cancer stage criteria, the 99 cases can also be classified as local advance (63.6%) and distant metastasis (36.4%).

The detection rate for left-sided tumors (5.5%) was significantly higher than that for right-sided tumors (3.5%) (*x* ^*2*^ = 4.923, *p* = 0.027). The metastasis detection rates differed depending on clinical stage classification (*x* ^*2*^ = 23.952, *p* < 0.001) (Table 4).

**Table 4:** Detection rates of RCC local advance or distant metastasis by location and clinic stage.

Group	Number of local advance or distant metastasis (%)		*x* ^*2*^	*p*
	Yes	No		
**Location**				
Left	62 (5.5)	1061 (94.5)	4.923	0.027
Right	36 (3.5)	987 (96.5)		
Total	98 (4.6)	2048 (95.4)		
**Clinical stage**				
T1a	16 (2.4)	648 (97.6)	23.952	0.000
T1b	13 (2.7)	466 (97.3)		
T2a	15 (9.7)	140 (90.3)		
T2b	6 (7.8)	71 (92.2)		
Total	50 (3.6)	1325 (96.4)		

Note: Because the location was missing for some cases with recorded clinical stage data, some groups have been combined.

According to Spearman’s correlation analysis, the metastasis detection rate was negatively correlated with tumor location (*r* = -0.048, *p* = 0.027), i.e. local advance or distant metastasis was more often in the left kidney. A positive correlation was also found between the metastasis detection rate and the size of tumor (*r* = 0.096, *p* = 0.000), with the likelihood of local advance or distant metastasis increasing with tumor size.

Using local advance or distant metastasis as the dependent variable (*Y*) and the possible influencing factors as independent variables (*x*), binary classification logistic regression analysis was performed. The results from Logistic regression indicated tumor size as a risk factor for metastasis (α = 0.05, β = 0.10, OR = 1.724). The risk of local advance or distant metastasis in the left kidney was 1.6-fold greater than that of the right kidney (Table 5).

**Table 5:** Factors affecting local advance or distant metastasis: binary classification logistic regression analysis.

Factors	*b*	*SE(b)*	*Waldc2*	*df*	*P*	*OR*	*OR95%CI*	
*x* 1 (Location)	-0.471	0.214	4.844	1	0.028	0.624	0.410	0.950
*x* 2 (Tumor size)	0.545	0.141	14.859	1	0.000	1.724	1.307	2.275

### The trends in RCC pathological changes from 2005 to 2014

RCC cases showed an increasing trend from 2005 to 2014 (Table 6), i.e. from 21.1% by 2007 to 65.2% by 2014.

**Table 6:** Distribution of the 2154 cases by year.

Year	Number			Percentage (%)	Increase rate (%)
	Total	Male	Female		
2005	95	54	41	4.4	—
2006	162	111	52	7.5	70.51
2007	153	96	57	7.1	-0.05
2008	160	104	56	7.4	0.04
2009	195	119	76	9.0	21.87
2010	221	147	74	10.3	13.33
2011	236	148	88	10.9	0.07
2012	310	201	108	14.4	31.36
2013	296	175	121	13.7	-0.05
2014	326	206	120	15.2	0.10
Total	2154	1361	793	100.0	

**Fig 1:**
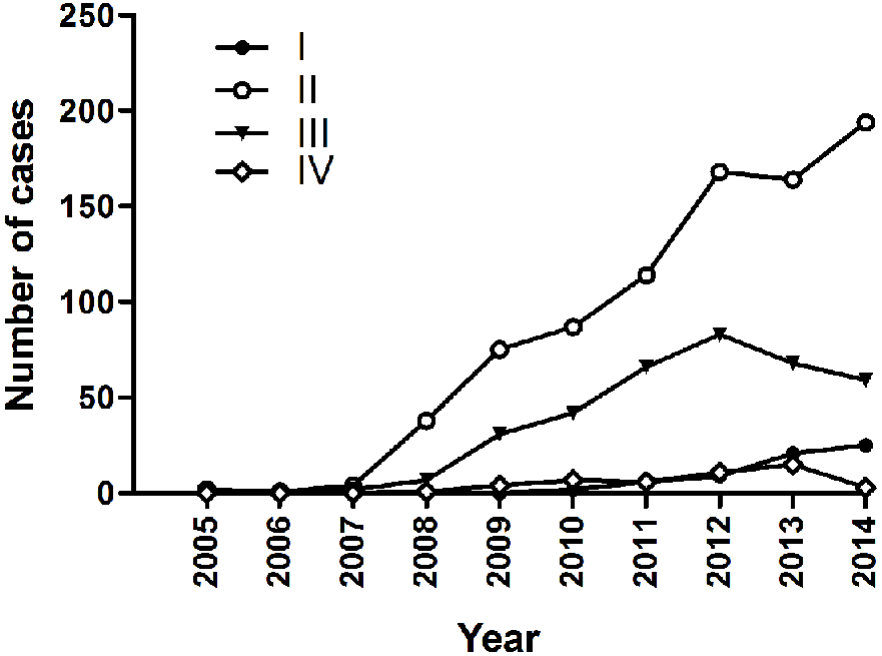
The change trend in increase of case number by Fuhrman grade from 2005 to 2014. In this figure, the X-axis represents the year from 2005 to 2014; the Y-axis represents number of the case. I: representing Fuhrman grade I; II: representing Fuhrman grade II; III: representing Fuhrman grade III; and IV: representing Fuhrman grade IV.

The number of case in Fuhrman grade I and grand IV do not increase significantly. The number of case in grade II and III showed an obvious increase. In addition, grade III seems to begin to goes down after 2012 (Fig 1).

The result from ANOVA indicated that the average tumor size decreased significantly over time (Table 7). ARIMA modeling of the time series data was used to predict the average RCC size, showing potential continual decrease from the first quarter of 2015 to the fourth quarter of 2016 (Table 8).

**Table 7:** Average tumor size by year.

Year	Number	Mean ± S.E.	*95%CI*	*F*	*P*
2008	40	5.957 ± 0.469	(5.009,6.905)	6.035	0.000
2009	153	6.039 ± 0.265	(5.515,6.562)		
2010	148	5.561 ± 0.285	(4.998,6.125)		
2011	198	4.982 ± 0.184	(4.620,5.345)		
2012	264	4.976 ± 0.161	(4.660,5.292)		
2013	274	4.973 ± 0.158	(4.662,5.285)		
2014	293	4.611 ± 0.140	(4.335,4.887)		
Total	1370	5.109 ± 0.075	(4.961,5.257)		

Note: The results from 2005 to2007 were not included in this Table due to the number of recorded tumor sizes was too few to carry statistic analysis.

**Table 8:** Average size of tumor prediction.

Time	Predicted value	95% CI
2015 first quarter	4.239	(3.018, 5.460)
2015 second quarter	4.156	(2.935, 5.377)
2015 third quarter	4.073	(2.852, 5.294)
2015 fourth quarter	3.990	(2.769, 5.211)
2016 first quarter	3.907	(2.686, 5.128)
2016 second quarter	3.824	(2.603, 5.045)
2016 third quarter	3.741	(2.520, 4.962)
2016 fourth quarter	3.658	(2.437, 4.878)

Note: This is from the first quarter of 2015 to the fourth quarter of 2016.

## Discussion

RCC is a kidney cancer that originates in lining of the proximal convoluted tubule, which is responsible for approximately 80–90% of malignant tumors of the kidney and accounts for 3% of all malignant tumors in adults [5]. Previous study from a large-scale population survey has indicated that the risk factors of RCC include long-term smoking, diet, obesity, hemodialysis, hypertension, and long-term antihypertensive use [6–14], indicating its complicated multi-factor origin, although the exact mechanisms remain unclear. The change trend of RCC differs in different regions worldwide. However there was no direct report regarding the pathologic characteristics and changes of RCC in a Chinese population. In this study, we have investigated and presented such data and the changes of RCC within the past 10 years in a representive population from Shanxi Province of China.

### The characteristics of demographic distribution and the increase of RCC incidence

RCC ranks as the 9^th^ most common tumor in males and the 14^th^ in females. According to relevant demographic studies, the RCC incidence has been increasing in most regions and populations of the world reported in many bodies of literature in past twenty years [1–3, 15–17]. In contract with this, the RCC incidence has remained steady or even decreased in some European countries such as Austria and Germany in recent years [18, 19]. The RCC incidence was reported in the lower level from 3.69 / 100,000 to 5.08 / 100,000 which accounts for 2–3% of all adult malignant tumors. However, the incidence of RCC shows a clearly increasing tendency, with a 2% increase, namely, adding 20,000 to 40,000 new cases per year [20]. For example, Ma et al. reported the increase tendency from 4.26 / 100,000 to 6.63 / 100,000 using registry reports from 11 cities of China over a period of 15 years [21]. According to present data from the three hospitals, the number of diagnosed patients in clinic visit showed an increasing trend in the cases of RCC, especially in the years of 2006, 2009 and 2012, which showed an increased rate by 70.52%, 21.87% and 31.36%, respectively. Although we fail to get the information of exact incidence during this period in this retrograde investigation, the tendency of increased case number in the present study of the past 10 years are consistent with other reports in China and globe data of increased incidence of RCC.

The RCC incidence clearly differs by region. Znar et al. compared more than 40 countries and regions, and found that the RCC incidences in the northern European countries are much higher than those in Asian and African countries. For example, Czech region has the highest RCC incidence (15 / 100,000), which is 15 times greater than that in the countries with the lowest RCC incidence [3]. In additional, event in a country, the RCC incidence may differ from area to area, for instance, the data from China’s Cancer Prevention and Control Office and the information center of the Health and Family Planning Commission show that the RCC incidence and mortality vary significantly in different areas in China as well. In general, the RCC incidence in urban areas is higher than that in rural areas [21, 22].

The RCC incidence in all five continents also shows that 63.3% out of the total increased cases is males in the year 2012[15]. Similarly, with 2,154 cases in our study, we found the ratio of the RCC case number in males verse to females was 1.71:1, which is in accordance with the above mentioned results worldwide and some other studies in China. For example, the data of the Tianjin from 1981 to 2001, cancer registry report show that the RCC incidence has clearly at accessional amplitude of 4.58% (male) and 3.20% (female) [23], namely, the incidence of RCC in females was approximately half of that of males. In addition, the present study also compared the gender difference of RCC pathological grade and found significant differences in their constituent ratios. The proportions of grades III and IV were bigger in males than that in females. Spearman’s test showed that the RCC pathological grade was negatively correlated to female which is consistent with the previous findings [7, 23]. Thus, it suggests that female is at a lower risk of RCC, with both in lower incidence and less severe in the RCC pathological grade. The exact mechanimsas are to be investigated in the later study.

Although RCC has been reported in all age groups, the risk of RCC increases with aging and the highest RCC incidence is at age of 50–70 [24–26]. In this study, we categorized all the subjects into 6 age groups, namely < 30, 30–40, 40–50, 50–60, 60–70, and > 70 years old, with the youngest age was 6 years, and the oldest 89 years. As a result, we observed that the greatest proportion (61.2%) of the total cases of the patients was in the age 50–70 years group as well. In summary, our finding indicates a similar tendency in the age distribution of RCC occurring in these populations.

### The clinicopathologica characteristics of RCC

Generally, the main histological subtypes of RCC according to the report of International Agency for Research on Cancer shows that the top three subtypes are clear cell renal cell carcinoma (CCRCC, 70%), papillary renal cell carcinoma (PRCC, 10%–15%), and chromophobe RCC (5%) [27]. In our study, the top three types were CCRCC (87.6%), PRCC (2.9%) and chromophobe RCC (2.8%), which was also supported by the similar studies in different regions of China [28, 29]. Compared with the reports elsewhere in the world, the subtype in this population trends to have bigger portion in CCRCC, which is different from that reported by International Agency for Research on Cancer.

Regarding tumor’s progress, we observed that around half (48.29%) of the total cases were in stage T1a, composing the largest proportion; 34.84% was in stage T1b as the second largest; the rest was stage T2a (11.27%) and T2b (5.60%). Interestingly, the longest RCC tumor diameter was found in the left kidney. So far as we know, no similar report was found in the literature. The reason remains unclear.

To reflect tumor prognosis, Fuhrman grading categorizes the renal cell nuclear into four grades based on its size, morphology, and nucleolus [30]. In 1315 cases with recorded Fuhrman grade in this study, 4.8% were grade I; 64.3% grade II; 27.4% grade III, and 6% grade IV. Significant differences were found in the average size of tumor among the pathological grades, i.e., the size of tumor increased with increasing RCC Fuhrman grade, with the linear relationship between pathological grades and the size of tumor. Actually, the tumor size and pathological grading are considered as a dynamic process, not only used as a tumor grading method but also a clinic indicator of malignancy [31] as a basis for making potential treatment plan for the patients with high-grade tumor. Whether they hold a potential role on disease progression for the patients with low-grade tumor is unclear for the time being. From a practical view, it worth explore its role of clinic application in the future. However, our study found that only 61% of the pathological files had recorded pathological grade, indicating the management is to be improved to keep an efficient file system.

### The changing trend in the size of RCC tumor from 2005 to 2014 and local advance or distant metastasis

In this study, a significant difference in the size of RCC tumor was found with time from year 2005 to 2014, namely, tumor size decreasing annually during this period, implicating that the majority of the RCC cases are inclined to become smaller. Therefore this population with small tumor size may become an important target of efficient therapy. To further describe the potential trend in tumor size, a time series prediction was carried on by using the data obtained in this investigation, the result shows that the average size of RCC tumor may continue decrease from the first quarter of 2015 to the fourth quarter of 2016.

Of the 2154 RCC cases, 4.6% exhibited local advances or distant metastasis, 63.6% of them belong to local advance and to the rest were distant metastasis. The relevance of the tumor size to RCC local advance or distant metastasis has been suggested by some literatures previously. A cohort study showed big tumor is inclined to metastasis [32]. In agree with this, the study of tumor size and metastasis in RCC patients from 1971–2005 in Iceland found that metastasis risk increased with increasing tumor size, but no linear relationship was found [33]. In this study, our result provides a support that the risk of local advance or distant metastasis increases with increased tumor size. However, whether reduce in the size of RCC tumor will result in a lower risk of local advance or distant metastasis needs further investigation.
